# The effects of continued obesity on the cardiovascular system

**DOI:** 10.1186/1532-429X-11-S1-P62

**Published:** 2009-01-28

**Authors:** Oliver J Rider, Jane M Francis, Steffen E Petersen, Stefan Neubauer

**Affiliations:** University of Oxford, Oxford, UK

**Keywords:** Abdominal Aorta, Left Ventricular Mass, Left Ventricular Diastolic Function, Ventricular Diastolic Function, Fasting Serum Glucose

## Objective

To determine the effects of a one year period of sustained obesity on the cardiovascular system

## Background

Although the effects of obesity and weight loss on the cardiovascular system have attracted a lot of interest, the effects of continued obesity are not well understood. Obesity is characterized by left and right ventricular hypertrophy and cavity dilatation, left ventricular diastolic dysfunction and reduced aortic elastic function, and weight loss is associated with at least partial reversal of these adaptive changes. Here, we present data looking at the effects of one year of continued obesity in seven obese subjects who were unsuccessful in losing weight over a dietary trial period.

## Methods

Seven obese subjects with no identifiable cardiovascular risk factors underwent CMR scanning before and after a period of one year of sustained obesity. All subjects underwent cardiac MR imaging at 1.5 T for the assessment of left ventricular mass (g), left ventricular end-diastolic volume (EDV; ml), stroke volume (SV; ml) and LV EF (%). Aortic distensibility was assessed at three levels; the ascending (Ao) and proximal descending aorta (PDA) at the level of the pulmonary artery and the abdominal aorta (AA). The abdominal cine images were piloted perpendicular to the orientation of the abdominal aorta. In addition to this, left ventricular diastolic function was assessed using volume time curve analysis.

## Results

**Figure 1:**
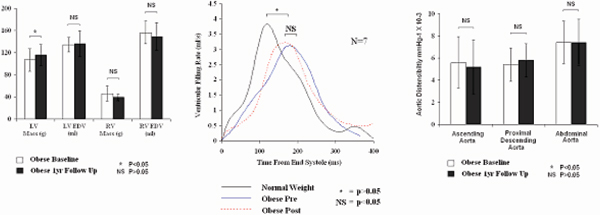
Figure 1

After one year there was no significant change in body mass index, weight, visceral fat mass or total fat mass. There were no significant differences in fasting serum glucose, serum cholesterol or systolic blood pressure. After one year of continued obesity, there was a significant 7% increase in left ventricular mass, and left ventricular mass when indexed to both height and height^2.7^. Right ventricular mass was unchanged over the year period. Both left and right ventricular end-diastolic volume, end-systolic volume and stroke volume were unchanged with one year of continued obesity. Left ventricular ejection fraction was also unchanged over the one year period. There was no significant difference between left ventricular diastolic function or aortic distensibility measurements taken at any level of the aorta before and after one year of continued obesity. Figure 1.

## Conclusion

In this small sub analysis we have shown that continued obesity over a period of one year results in increases in left ventricular mass without any other change in ventricular or aortic function. This again points to the fact that left ventricular mass is very sensitive to body mass index and, in the setting of obesity, can increase or decrease over a period of one year.

